# Telethon Network of Genetic Biobanks: a key service for diagnosis and research on rare diseases

**DOI:** 10.1186/1750-1172-8-129

**Published:** 2013-08-30

**Authors:** Mirella Filocamo, Chiara Baldo, Stefano Goldwurm, Alessandra Renieri, Corrado Angelini, Maurizio Moggio, Marina Mora, Giuseppe Merla, Luisa Politano, Barbara Garavaglia, Lorena Casareto, Francesca Dagna Bricarelli

**Affiliations:** 1 UOSD Centro di Diagnostica Genetica e Biochimica delle Malattie Metaboliche, Istituto G. Gaslini, Largo G. Gaslini 5, 16147 Genova, Italy; 2 SC Laboratorio di Genetica Umana, E.O. Ospedali Galliera, Genova, Italy; 3 Centro Parkinson, Istituti Clinici di Perfezionamento, Milano, Italy; 4 UOC Genetica Medica, Dipartimento di Biotecnologie Mediche, Università di Siena e Azienda Ospedaliera Universitaria Senese, Siena, Italy; 5 Dipartimento di Neuroscienze SNPSRR, Università di Padova, IRCSS San Camillo, Venezia, Italy; 6 UOD Diagnostica Malattie Neuromuscolari e Rare, Fondazione IRCCS Ca’ Granda Ospedale Maggiore Policlinico, Milano, Italy; 7 Laboratorio di Biologia Cellulare, UO Malattie Neuromuscolari e Neuroimmunologia, Fondazione IRCCS Istituto Neurologico C. Besta, Milano, Italy; 8 Unità di Genetica Medica, IRCCS Casa Sollievo della Sofferenza, S. Giovanni Rotondo (FG), Italy; 9 Cardiomiologia e Genetica Medica, Dipartimento di Medicina Sperimentale, Seconda Università di Napoli e Azienda Ospedaliera Universitaria SUN, Napoli, Italy; 10 UO Neurogenetica Molecolare, Fondazione IRCCS Istituto Neurologico C. Besta, Milano, Italy; 11 Ufficio Coordinamento Network, c/o UOSD Centro di Diagnostica Genetica e Biochimica delle Malattie Metaboliche, Istituto G. Gaslini, Genova, Italy; 12 Dipartimento Ligure di Genetica, c/o E.O. Ospedali Galliera, Genova, Italy

**Keywords:** Biobanking, Networking, Biological resources centre, IT infrastructure, Biological material, Biospecimens, Cryopreservation, Rare diseases, Patients’ associations

## Abstract

Several examples have always illustrated how access to large numbers of biospecimens and associated data plays a pivotal role in the identification of disease genes and the development of pharmaceuticals. Hence, allowing researchers to access to significant numbers of quality samples and data, genetic biobanks are a powerful tool in basic, translational and clinical research into rare diseases. Recently demand for well-annotated and properly-preserved specimens is growing at a high rate, and is expected to grow for years to come. The best effective solution to this issue is to enhance the potentialities of well-managed biobanks by building a network.

Here we report a 5-year experience of the Telethon Network of Genetic Biobanks (TNGB), a non-profit association of Italian repositories created in 2008 to form a virtually unique catalogue of biospecimens and associated data, which presently lists more than 750 rare genetic defects. The process of TNGB harmonisation has been mainly achieved through the adoption of a unique, centrally coordinated, IT infrastructure, which has enabled (i) standardisation of all the TNGB procedures and activities; (ii) creation of an updated TNGB online catalogue, based on minimal data set and controlled terminologies; (iii) sample access policy managed via a shared request control panel at web portal. TNGB has been engaged in disseminating information on its services into both scientific/biomedical - national and international - contexts, as well as associations of patients and families. Indeed, during the last 5-years national and international scientists extensively used the TNGB with different purposes resulting in more than 250 scientific publications. In addition, since its inception the TNGB is an associated member of the Biobanking and Biomolecular Resources Research Infrastructure and recently joined the EuroBioBank network. Moreover, the involvement of patients and families, leading to the formalization of various agreements between TNGB and Patients’ Associations, has demonstrated how promoting Biobank services can be instrumental in gaining a critical mass of samples essential for research, as well as, raising awareness, trust and interest of the general public in Biobanks. This article focuses on some fundamental aspects of networking and demonstrates how the translational research benefits from a sustained infrastructure.

## Background

Genetic biobanks (GBs) have long been a powerful tool in basic, translational and clinical research, and in care practice of rare diseases: indeed GBs allow researchers the access to significant numbers of quality samples and associated data. Recent advances in the technology of molecular biology and genetics, dramatically increasing the demand for well-annotated and properly preserved specimens, have contributed to raise the awareness of the importance of coordinated biobanking activity. The best and the most effective solution to that demand is to enhance the potentialities of well-managed biobanks by building a network.

Here we report a 5-year experience of the Telethon Network of Genetic Biobanks (TNGB), a non-profit organization of Italian repositories created to form a unique catalogue of biological samples and associated data presently listing more than 750 rare genetic diseases.

The need for networking was recognised in 2008 by one of the biggest biomedical Italian charity always committed to the study of genetic diseases, Telethon Foundation (TF), and was justified by the growing demand for biological material to develop studies in rare genetic disorders. Hence the network was established to (i) centralise very rare samples and data, (ii) minimise biases potentially arising from heterogeneity in the quality of samples by developing standard procedures and common quality assurance policies, (iii) enhance collaboration inside the biomedical community, and more importantly (iv) promote biobanks within Patients’ Associations, foster their active participation and share benefits with them.

The TNGB initiative was also based on the fact that Telethon had financially supported some genetic biobanks, as single core facilities, since 1993. Therefore, TNGB membership was initially limited to the seven well-managed GBs, but was expanded, over 5-year project, to other 3 biobanks selected through specific calls. As the profiles of the TNGB partners were diverse in their data management, models and policies, one of the priority goals of TNGB has been to harmonise a functional net from pre-existing biobanks by setting out common rules defining network’s ethical, legal and societal policies as well as standard operating procedures. TNGB has developed and shared a high-powered IT infrastructure to standardise and harmonise sample collection and data annotation, as well as to build a web site to facilitate researcher access and improve TNGB worldwide visibility. Indeed, although each GB locally operates in independent way, the databases of all partners are linked and accessible via a central web portal.

This article therefore reports some basic aspects of networking, including governance, management and IT frameworks to facilitate the establishments of best practice and standardizations and to ensure that the interconnected biobanks are acting within their remit, as well as within the national/international laws, regulations and recommendations. In addition, the specific experience of TNGB in the field of rare-disease-biobank networking also demonstrates how the translational research benefits from a sustained infrastructure.

## TNGB composition

**Figure 1:**
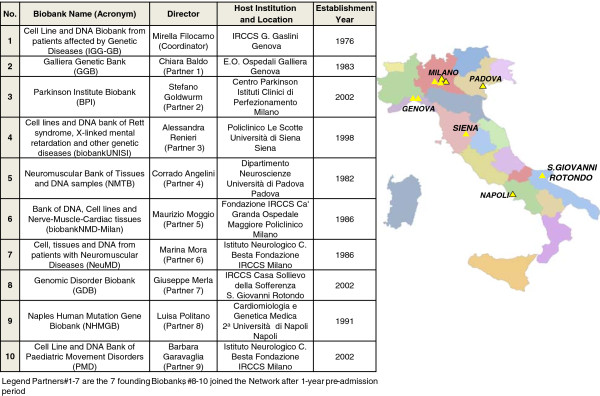
**Geographical location of the genetic biobanks in Italy.** Yellow triangle denotes biobanks, the black outline marks the “Joint Neuromuscular Biobanks” subgroup. Composition and institutional affiliation of the Biobanks are reported in the annexed schema on the left.

Currently the network is composed of 10 Biobanks geographically located in different areas of Italy: while the founding Biobanks of the Network are seven, the 3 others joined the Network after 1-year pre-admission period required for adopting and implementing all the procedures. Because of the peculiarity of the stored pathologies, 4 biobanks are part of the so called “Joint Neuromuscular Biobanks” subgroup. The composition of TNGB is depicted in Figure 1 and the main characteristics of the individual Biobanks are detailed in the annexed schema. It is notable that 6 out of 10 Biobanks have been established in the 70-80’s with the oldest in 1976 and the youngest in 2002, though. Another valuable aspect of TNGB composition is that the Host Laboratories, Departments and Institutions, where the 10 Biobanks have been established, have a longstanding tradition and internationally recognised expertise in the diagnosis of, and research into, rare genetic diseases. Hence, a combination of various activities, supported by skilled clinicians, pathologists, biochemists and geneticist, has allowed building, over the course of several years, these Biobanks preserving well-documented biospecimens and associated data, which can also be continuously updated on the basis of both clinical revisions and more recent scientific acquisitions.

## TNGB governance

The TNGB is governed by the Network Board (NB), the decision-making body composed of the Coordinator and Biobank Directors. Basically, the NB is involved in defining the strategic orientation of the Network and establishing the annual work plan.

The Advisory Board (AB) includes some external members, with expertise in legal, ethical and technical issues; its composition can change over time depending on Network activity evolution.

Telethon Foundation (TF), the funding body, has checked that the project run smoothly. The assessment of both TNGB and all funded entities, carried out by the Telethon Scientific Committee for Biobanks, is based on annual reports summarising activities and accomplishments.

The Coordinator, elected for the whole duration of the grant by the NB among the Biobank Directors, plays a central role and maintains the contacts with the Network Board, the Advisory Board and the Telethon Scientific Office. The Coordinator is supported by a Coordinator Emeritus having the main tasks of promoting the Network and interacting with Patients’ Associations.

**Figure 2:**
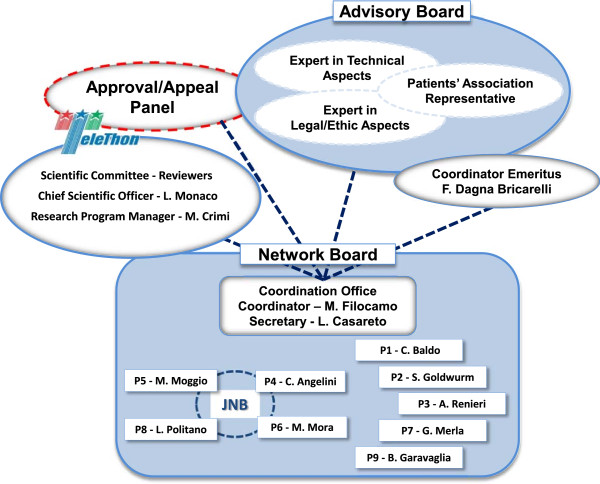
**Graphical representation of the TNGB governance bodies.** Legend: P = Partner; JNB = Joint Neuromuscular Biobanks.

In case of particular controversies, an external committee, the Approval/Appeal Panel (AP), can be convened for a third-party opinion. Typically, the panel composition includes one AB member, one from the Telethon Biobank Committee, and a third external member selected among the International Scientific Community. The AP composition is concerted with TF, in accordance with peer-review-based procedures, and depends on the type of the controversial issue. Figure 2 is a graphical representation of the main TNGB governance bodies.

Relationships among the TNGB partners including rules for decision-making processes, ethical guidelines, activities, policies, expected benefits, and undertaken duties have been laid down in the TNGB Charter [1].

### Harmonisation and standardisation: the role of the coordination office

The coordination office (CO) is involved with day-to-day operational aspects to guarantee a harmonised management of the Network through the standardisation of all the main activities.

Standard operating procedures (SOPs) for sample collection, processing and storage employed across the TNGB Partners have been shared with only minor local modifications and made available on the TNGB web site. Standardisation has been mainly achieved through database management, minimal data set, controlled terminologies, sample access policy, common informed consent form, and quality control tools aimed at identifying potential bias due to sample processing as well as variables unrelated to the disease being studied. Harmonisation of policies, SOPs and documents is however an ongoing activity: indeed, all documents are continuously reviewed to be compliant with both national and European guidelines and directives.

Importantly, by promoting daily interoperability amongst the TNGB’s members, CO has also avoided inefficiencies potentially arising from individual initiatives.

## IT infrastructure

The IT infrastructure, developed by SoftWerk (Genova, Italy) [2], is the management tool of the network. Each TNGB Partner has been provided with a small server machine with a free operating system (GNU/Linux) with open source software (LAMP stack solution) and preinstalled web-like applications accessible to the TNGB members regardless of their operating system. The servers provide local storage and backup; in addition, to execute schedule off-site backups and to feed data to the central database consolidator, they are connected to the central network servers by virtual private network (VPN) techniques.

Hence, sharing an IT platform has not meant a loss in autonomy as the associated biobanks, residing within their own host institution, manage the core biobank data by optional modules adapted to needs and peculiarities of each biobank. The IT system automatically aggregates from each local database a minimum coded dataset which is published on the TNGB web site in a single online catalogue.

Data safety is guaranteed by server redundancy and automated cyclic on-site, off-site and cross-site encrypted backups both on optical media and remote servers through Secure Socket Layer (SSL) 128 bit encrypted channels.

### Web access to the network

The Network has published a web site [3] which provides access to the public pages, the Network’s intranet and the Request Control Panel.

The public area contains general network information and contacts, online aggregated catalogue and a search engine for the aggregated biobank data, login reserved area, TNGB official documents (The Charter, Biobank guidelines, SOPs), distinct agreements between TNGB and Patients’ Associations, and list of publications acknowledging TNGB services.

## TNGB: samples and pathologies

**Figure 3:**
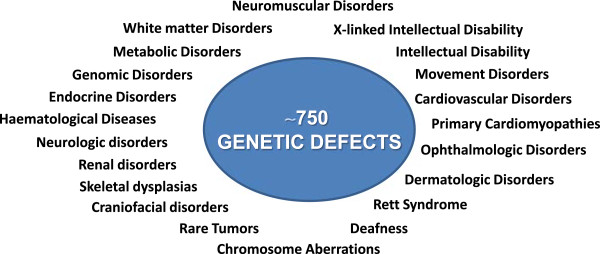
Classification of the diseases into large subgroups.

The Biobanks of the Network collectively preserve 75,900 biospecimens deriving from more than 750 different genetic defects. As shown in Figure 3, a concise classification of the diseases includes: Cardiovascular disorders; Chromosome aberrations; Craniofacial disorders; Deafness; Dermatologic disorders; Endocrine disorders; Genomic disorders; Haematological diseases; Intellectual disability; X-linked intellectual disability; Metabolic disorders; Neuromuscular disorders; Neurologic disorders; Movement disorders; Ophthalmologic disorders; Primary Cardiomyopathies; Rare Tumors; Renal disorders; Rett Syndrome; Skeletal dysplasias; White matter disorders.

**Figure 4:**
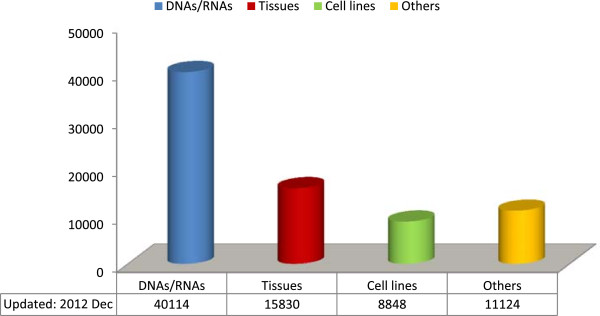
**TNGB biospecimen typologies.** Legend: Others = blood, leucocytes in DMSO, T-lymphocytes + il-2, liquor, serum/plasma. Tissues = muscle, nerve, skin, foetal tissues. Cell lines = fibroblasts, EBV-lymphoblasts, lymphocytes, myoblasts, amniocytes, trophoblast cells, iPS.

Figure 4 reports the main types of biospecimens, stored in the Biobanks, which include foetal and adult cell lines (amniocytes, trophoblast cells, fibroblasts, myoblasts, lymphoblasts and T-lymphocytes activated with IL-2), peripheral blood lymphocytes, muscle and nerve tissues, tissues derived from foetal loss, DNA/RNA samples, sera/plasma and whole blood samples, and iPS cells.

## Sample management

### Storage

The *incoming samples* are managed by each partner and locally recorded in the respective individual databases. The samples will be processed for the storage only with the written informed consent of the patients or their parents or legal guardian in cases where an individual is considered unable to give consent. In addition, a material transfer agreement (MTA-in), signed by the referring clinician, should accompany each sample and include at least the minimum TNGB-shared data set, that is donor’/patient’s generalities (name, date of birth, address, ethnic origin, gender), phenotype (affected/not affected), essential anamnestic data (presence of consanguinity and/or familiarity, tissue and/or organ anomalies, laboratory test anomalies, etc.), diagnosis data (modality, centre performing diagnosis), sample data (code, type, data of collection, etc.).

To ensure a uniform codification and classification, the diseases of the catalogue are defined by OMIM (Online Mendelian Inheritance in Man) number, Orpha (Orphanet classification of Diseases) number, and ICD (International Classification of Diseases) code.

### Sample access policy

Basic rules to manage access to collections and related data are shared among the TNGB partners and applied to all researchers, including the Biobank Staff. General criteria include: (i) guarantee that an adequate aliquot of sample be saved for patients and/or their relatives, aimed at potentially retrospective analyses; (ii) sample distribution only to qualified professionals working at research or medical institutions engaged in health-related research or health care; (iii) appropriate justification for sample use; (iv) sample transfer only if the material transfer agreement form has been signed from Principal Investigator; (v) project design in agreement with TNGB mission and policies.

TNGB has also recently implemented and adopted a cost recovery system aimed at making biobanks financially semi-independent. Briefly, researchers are requested to partially cover the cost of some basic procedures related to the TNGB distribution services, in addition to the shipping costs.

**Figure 5:**
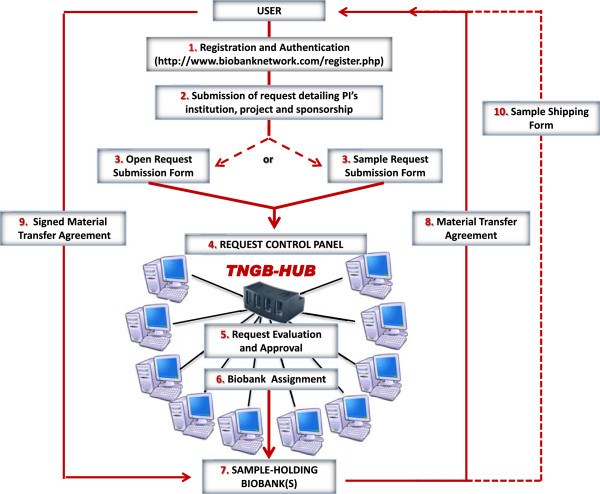
Policy for sample distribution.

As shown in Figure 5, the procedure of *outgoing samples* occurs entirely through TNGB web portal: for requesting samples, the users, including Biobank staff, must first register at the relevant web-page. After authentication, users can submit their request filling in the online “Request submission form”. The submission implies that users provide some essential information including a brief description of the project and research-grant sponsor. Users can select samples from the catalogue as a specific diagnosis classified according to OMIM, Orpha, ICD codes, or alternatively, by configuring a query for some their attributes such as type of sample, gender, phenotype, type of diagnosis, etc. Alternatively, users can submit the request by describing the main characteristics of the samples necessary for their study. The so called “open request” implies that users will be supported by the relevant Biobank Staff to select the most suitable samples. Once the request is centrally submitted at the Request Control Panel (RCP) and has obtained the consensus of all Network Board, the coordination office passes it on the sample-holding biobank’s Staff, who replies with a proper Material Transfer Agreement form (MTA-out). The assignment of the concerned Biobank(s) requires at the latest 5 working days for consistent requests. By signing this form the user undertakes to respect the TNGB’s rules and policies. The main MTA-out issues include: scientific manager; project and research sponsor; type and quantity of concerned samples; citation in acknowledgements; transfer of samples to third parties; communication of scientific results; cost recovery and shipping cost.

The RCP access, protected by password, is available for Biobank Directors who can share, process, monitor and update all TNGB requests.

The RCP is, therefore, an innovative tool which has put into practice the harmonisation process through the management and the monitoring of all TNGB requests. Most importantly, RCP has also avoided duplication of research efforts on very rare and precious biospecimens.

**Figure 6:**
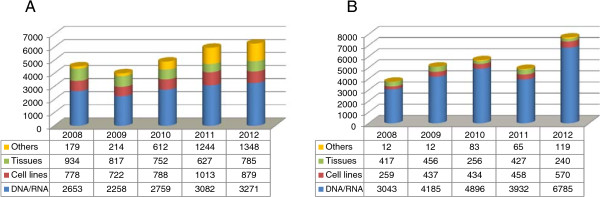
**Sample workflow.** The graphics illustrate the incoming **(A)** and outgoing **(B)** flow of the TNGB samples. Legend: Others = blood, leucocytes in DMSO, T-lymphocytes + il-2, liquor, serum/plasma. Tissues = muscle, nerve, skin, foetal tissues. Cell lines = fibroblasts, EBV-lymphoblasts, lymphocytes, myoblasts, amniocytes, trophoblast cells, iPS.

Figure 6 reports details concerning the large amount of the incoming and outgoing samples from 2008 to 2012. In particular, as shown in the diagram on the left (Figure 6A), a total of 25,712 samples and data have been biobanked with an annual average of 5,142 (ranging from 4,010 to 6,283). In the same vein, the outgoing sample volume had a clear tendency to increase (Figure 6B), being totally 27,086 with an annual average of 5,417 ranging from 3,731 to 7,714. To be also noted the significant increase of the variety of the incoming samples (Figure 6A) which reflects a Network particularly careful to satisfy current and future demands of the genomic and proteomics studies requiring vast amounts of samples.

### Request typology

Over the past 5 years, national and international scientists have extensively used the TNGB with different purposes, as well as, patients and patients’ family members have been able to rely on the TNGB services.

The activities related to this service can be broken into three main categories:

1) “research”: in this field the Network supported 784 research projects by providing several thousand of samples;

2) “diagnosis”: this category refers to a service that is an added value of the TNGB as it provides access to clinicians who require storing samples from “undiagnosed” patients aimed at future diagnoses. In this field the TNGB fulfilled a total of 441 requests even though a considerable number of samples from patients without diagnosis still remains in each Biobank;

3) “family”: TNGB has also supported members from 18 families at risk for a rare disease. These users, being aware of the Biobank storing the sample(s) from the respective patients’ index case, directly contacted therefore the concerned Biobank and requested sample(s) to be used for genetic counselling including the prenatal diagnosis.

### Return of research findings

**Figure 7:**
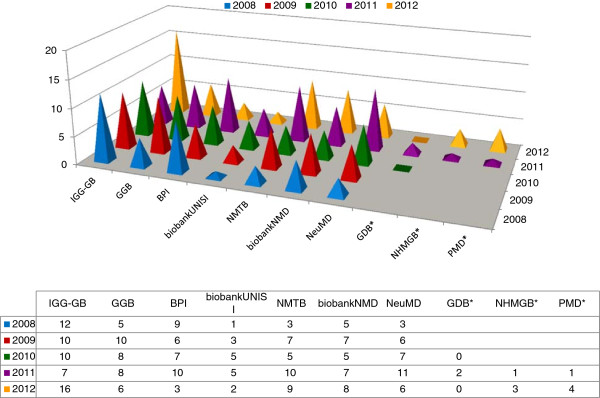
**Scientific production resulting from the 10 Biobanks’ services.** Legend: asterisk denotes that the Biobank joined the network in 2010 (GDB) and 2011 (NHMGB, PMD), respectively**.** To be noted that the Biobank acronyms are defined in the annexed schema of Figure 1.

Published and/or non-published analytical results obtained using the Biobank samples are gathered through the relevant “Confirmation of sample use” form periodically sent to TNGB users. During the 5-year period more than 250 scientific publications resulted from research conducted thanks to the services provided by the 10 Biobanks, which were formally acknowledged in the proper section of the related manuscripts, accordingly [4]. Figure 7 is the graphical representation of the scientific production resulting from the distribution service by Biobank.

By using TNGB samples, impressive results were achieved in the field of rare diseases and, whenever of clinical interest, the priority was to promptly return them to the patients/donors. The main findings included: discovery and/or characterisation of new syndromes; new gene identification; molecular and functional studies; epidemiologic studies; method set up; genotype/phenotype correlation studies; therapeutic studies; stem cells and clinical applications. Retrospective diagnoses were also reached in several patients. Additional file 1: Table S1 reports some significant examples per result-category for each Biobank and shows how patients and, by extension, the healthcare have benefited from the research outcomes conducted with the TNGB samples.

## Ethical, legal and social issues (ELSI)

Given the quantitative and qualitative value of the information attached to the biobank’s sample, it is imperative that TNGB protects the patients/donors’ confidentiality according to national and international regulations and recommendations [5-10]. In the same vein, the possibility of tracing the sample back to its patient/family/donor is also essential in the event of scientific results of use to the donor. Therefore, the registration of the samples is performed in identifiable mode, which means coding biological material for research purposes but making the link to their source possible through the use of a code known to Biobank Director and authorised staff only.

### Informed consent

Consent form and patient information sheet, shared by TNGB to seek the permission for biobanking from the donors or their legal representative, disclose all aspects related to the handling of the samples and data including: (i) sample usage: it is made clear that the sample may be used for further investigations of an exclusively diagnostic and/or research nature in the field of the concerned pathology only, and never for direct profit; if the sample is to be used in a different project a new consent will be sought; (ii) results: it is explained how the potential benefits derived from the use of the sample can positively impact upon the health of the individual and/or the entire community; (iii) confidentiality: the procedures for handling the data to ensure protection of privacy are illustrated; (iv) service guarantee: it has to be clarified that while handling and storage of the biological material are the responsibility of the Biobank Director, other accidental damage to the sample can unpredictably occur; (v) consent withdrawal: it is made explicit that consent can be withdrawn at any time removing the sample and relevant information. These aspects were also in line with other studies. [11].

The subject who has received the information has the possibility to take separate decisions regarding whether or not to (i) authorise the preservation; (ii) authorise the use of sample for scientific research; (iii) wish to be informed about the results deriving from continuing research.

After signing the consent form, the subject receives a copy countersigned by the person responsible for the Biobank as a guarantee of the respect for the statements.

Concerning the samples stored in the past without informed consent, in accordance with ESHG (European Society of Human Genetics) document [12], they are used in an identifiable manner guaranteeing confidentiality according to the rules of professional deontology and existing regulations, without the obligation to render the samples anonymous, in order to be able to make any diagnosis and/or important result available to biological patients’ family members.

## Patients’ Associations

The TNGB has developed a close relationship with Patients’ Associations since its inception: indeed, their representative has always been active part of the Advisory Board. This has enabled them to be involved in the drafting of policies and procedures for the improvement of the TNGB infrastructure including ethical issues such as transparency, consent, privacy, confidentiality, use and transfer of samples.

Alongside this, several meetings and workshops have been organised with the main aim to raise awareness, trust and interest in Biobanks as well as to introduce patients and their families to the concept of this bioresource as an effective service for collecting and centralising rare samples for specific research projects. In this respect, the Coordinator Emeritus has played a major role with the active collaboration and support of UNIAMO [13] (Italian Federation of about 100 Associations of patients with rare diseases) and other national Associations. The involvement of patients and families has proved to be instrumental in both gaining a critical mass of samples, that is essential for research into very rare diseases [14], and ensuring that patients’ needs and expectations in the field of biobanking be taken into due consideration.

Through this dissemination activity at national level, the interest in the Biobanks’ services is enormously increased among patients and their families. In fact, a framework agreement has been formalised between Telethon and UNIAMO to be the basis of each agreement between one of the Biobanks of the Network and a specific Patients’ Association. Currently, 6 agreements [15] have been signed with the first one formalised in 2009 and annually confirmed up to now.

**Figure 8:**
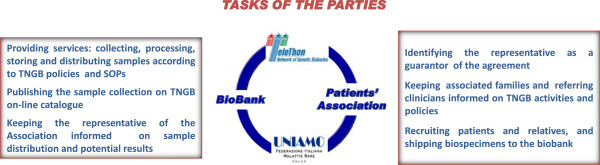
Agreement with patients’ association: tasks of the parties.

To our knowledge, this type of agreement, defining rules and tasks of the parties, viz. Genetic Biobanks and Patients’ Association, is unique at national and international levels and aims at supporting families with biobanking in a harmonized environment that ensures quality and proper use of the samples, as well as individuals’ confidentiality throughout the entire process (Figure 8). But, more importantly, patients’ samples, being listed in the TNGB catalogue, are publicly visible to the researchers who visit the web page. Another equally important aspect is that the Associations can play an active part within this framework agreement by promoting as well as cofounding specific projects on their collections. In this respect, “Ring 14 Association”, the first to sign the agreement in 2009, has currently two ongoing projects.

## Final considerations

The results of first 5 years of TNGB activities have confirmed the validity of this management model and more importantly showed that the Telethon’s investment policy in Biobanks, started in 1993, has so far been effective.

The Network is continuing to expand centralising a broader range of very rare disease types and recently partnering with EuroBioBank [16] which has been the first European network of genetic biobanks of rare diseases. In addition, since its inception TNGB, fulfilling the requested quality criteria, joined the pan-European research infrastructure BBMRI [17] as associated member. Within this framework, the Network also was one of the ten prototypes selected by Technopolis Group for a short/long term impact analysis, commissioned by BBMRI, aimed to support discussions on the sustainability of the future BBMRI research infrastructure [18]. Currently, TNGB is actively involved in the constitution of the Italian Node (BBMRI-IT).

The qualifying aspects of the TNGB can be summarised as follows: (i) the rigorous selection criteria adopted by Telethon for choosing the Biobanks to receive funding along with a thorough annual report to revise the budget: this method has been greatly stimulating and has provided the Biobanks direction for ameliorative actions to be taken in terms of services, quality management and information dissemination; (ii) the sensitivity of Telethon Scientific Office to deal with some of the issues raised over time: thanks to this *modus operandi* TNGB has achieved considerable progress and, at the same time, it has gradually developed a common culture about biobank governance system; (iii) the special attention paid to patients and families with genetic disorders: these concerted activities between TNGB, Telethon Scientific Office and Patients’ Associations have increased both the dissemination of knowledge about the Biobank key role among the community as well as the use of their services.

Recently TNGB has become one of the associated partners of the European RD-connect project aimed at connecting databases, registries, biobanks and clinical bioinformatics for rare disease (RD) research [19]. Therefore TNGB is engaged in the process of building a global harmonised infrastructure to efficiently distribute quality controlled samples and associated data for the study of RD in a protected ethical and legal framework. In connection with this, TNGB is also focused on improving services and quality management of each individual Biobank of the Network through the implementation of a certification program by applying International Organization for Standardization (ISO) 9001 and ISO 15189 which include specific indicators for Genetic Biobanks (“Standard criteria for quality management for Genetic Biobanks”, developed by Italian Society of Human Genetics [20]).

Concerning the future of TNGB, recent advances in next-generation sequencing technologies provide unprecedented opportunities for using the current Network to encompass a broader range of disease types. However, the potential of the new high-throughput genotyping technologies raises ethical issues not only including the consent that currently is not “broad” but restricted to that specific pathology of the patient, but also the management of the excessive amount of information, in particular of the so called “incidental findings” [21]. In consideration of this, legal and ethical aspects still need to be addressed. Based on a longstanding experience of TNGB, a collaboration with experts, including Italian data protection Authority, has started with the aim to accelerate the definition of national regulations for Genetic Biobanks.

## Competing interests

There are no financial or non-financial competing interests related to this manuscript.

## Authors’ contributions

MF coordinated the project and drafted the manuscript with the assistance of FDB and CB. All network partners (CB, SG, AR, CA, MMog, MMor, GM, LP, BG) helped to draft the manuscript. LC, as responsible for administrative activities, gave a substantial contribution throughout the project. The staff of the ten Biobanks was involved in the daily biobanking operations. All authors read and approved the final manuscript.

## Supplementary Material

Additional file 1: Table S1 Return of significant research findings: one example per result-category is reported for each Biobank. Click here for file

## Telethon Network of Genetic Biobanks Staff

Fabio Corsolini^1^;

Sara Galotto^1^;

Raffaella Mazzotti^1^;

Giorgia Stroppiana^1^;

Mauro Castagnetta^2^;

Massimo Mogni^2^;

Valeria Viotti^2^;

Alba Bonetti^3^;

Franca Felici^3^;

Francesca Natuzzi^3^;

Sonia Amabile^4^;

Elisa Frullanti^4^;

Ilaria Meloni^4^;

Marina Fanin^5^;

Annachiara Nascimbeni^5^;

Elena Pegoraro^5^;

Enrico Peterle^5^;

Laura Napoli^6^;

Michela Ripolone^6^;

Monica Sciacco^6^;

Raffaella Violano^6^;

Eleonora Canioni^7^;

Sara Gibertini^7^;

Simona Saredi^7^;

Simona Zanotti^7^;

Carmela Fusco^8^;

Lucia Micale^8^;

Maria Teresa Pellico^8^;

Leopoldo Zelante^8^;

Paola D’Ambrosio^9^;

Esther Picillo^9^;

Antonella Taglia^9^;

Chiara Barzaghi^10^;

Celeste Panteghini^10^;

Lorella Valletta^10^.
