# Supplementary material for: Telethon Network of Genetic Biobanks: a key service for diagnosis and research on rare diseases
Source: Orphanet J Rare Dis. 2013 Aug 30;8:129. doi: 10.1186/1750-1172-8-129 (PMC3766640; doi:10.1186/1750-1172-8-129)
Supplement: Additional file 1: Table S1 — Return of significant research findings: one example per result-category is reported for each Biobank. [file 1750-1172-8-129-S1.docx]

| **Table S1:**  **Return of significant research findings: one example per result-category is reported for each Biobank** | | | | | | | | |
| --- | --- | --- | --- | --- | --- | --- | --- | --- |
| **BIOBANK ACRONYM** | **RETROSPECTIVE DIAGNOSIS** | **DISCOVERY OF NEW SYNDROMES AND GENES** | **MOLECULAR AND FUNCTIONAL STUDIES** | **METHOD SET UP** | **GENOTYPE/ PHENOTYPE CORRELATION STUDIES** | **THERAPEUTIC STUDIES** | **GENETIC EPIDEMIOLOGIC STUDIES** | **STEM CELLS AND CLINICAL APPLICATIONS** |
| IGG-GB | Biancheri et al.  [1] | Hennies et al.  [2] | Chiefari et al.  [3] | Fuccio et al.  [4] | Tappino et al.  [5] | Porto et al.  [6] | Santamaria et al.  [7] | *Caiazzo et al.  [8] |
| GGB | Nikkel et al.  [9] |  | De Vita et al.  [10] | Sessarego et al.  [11] | Colombo et al.  [12] | Passerini et al.  [13] | Madia et al.  [14] | Teodolinda et al.  [15] |
| BPI | Cilia et al.  [16] | Lautier et al.  [17] | *Cartelli et al.  [18] |  | Healy et al.  [19] |  | Höglinger et al.  [20] | *Liu et al.  [21] |
| biobankUNISI | Ariani et al.  [22] | Ariani et al.  [23] | Makrythanasis et al.  [24] | Mencarelli et al.  [25] | De Filippis et al.  [26] |  | Bijlsma et al.  [27] | Amenduni et al.  [28] |
| NMTB | Fanin et al.  [29] | Torella et al.  [30] | Nascimbeni et al.  [31] | Spinazzi et al.  [32] | Nascimbeni et al.  [33] | Angelini et al.  [34] | Fanin et al.  [35] |  |
| biobankNMD | Mancuso et al.  [36] | Kornblum et al.  [37] | Ronchi et al.  [38] | Ronchi et al.  [39] | Magri et al.  [40] | De Palma et al.  [41] | Mercuri et al.  [42] |  |
| NeuMD | Saredi et al  [43] | Manzini et al.  [44] | Cesana et al.  [45] | Zanotti et al.  [46] | Mercuri et al.  [47] | Incitti et al.  [48] | Guglieri et al.  [49] | Tedesco et al.  [50] |
| GDB | *Micale et al.  [51] |  |  |  | *Ejarque et al.  [52] |  |  |  |
| NHMGB | *Cacciottolo et al.  [53] |  | Viggiano et al.  [54] |  | *Bello et al.  [55] |  |  |  |
| PMD | Panteghini et al.  [56] | Haack et al.  [57] | Campanella et al.  [58] | Leoni et al.  [59] | Chiapparini et al.  [60] |  | *Santorelli et al.  [61] |  |
| * Asterisk denotes manuscripts resulting from service provided by more than one Biobank | | | | | | | | |

**Table S1 References**

1. Biancheri R, Zara F, Rossi A, Mathot M, Nassogne MC, Yalcinkaya C, Erturk O, Tuysuz B, Di Rocco M, Gazzerro E, Bugiani M, van Spaendonk R, Sistermans EA, Minetti C, van der Knaap MS, Wolf NI: **Hypomyelination and congenital cataract: broadening the clinical phenotype.** *Arch Neurol* 2011, **68:**1191-1194.
2. Hennies HC, Kornak U, Zhang H, Egerer J, Zhang X, Seifert W, Kühnisch J, Budde B, Nätebus M, Brancati F, Wilcox WR, Müller D, Kaplan PB, Rajab A, Zampino G, Fodale V, Dallapiccola B, Newman W, Metcalfe K, Clayton-Smith J, Tassabehji M, Steinmann B, Barr FA, Nürnberg P, Wieacker P, Mundlos S: **Gerodermia osteodysplastica is caused by mutations in SCYL1BP1, a Rab-6 interacting golgin.** *Nat Genet* 2008, **40:**1410-1412.
3. Chiefari E, Iiritano S, Paonessa F, Le Pera I, Arcidiacono B, Filocamo M, Foti D, Liebhaber SA, Brunetti A: **Pseudogene-mediated posttranscriptional silencing of HMGA1 can result in insulin resistance and type 2 diabetes.** *Nat Commun* 2010, **27:**1-40.
4. Fuccio A, Iorio M, Amato F, Elce A, Ingino R, Filocamo M, Castaldo G, Salvatore F, Tomaiuolo R: **A Novel DHPLC-Based Procedure for the Analysis of COL1A1 and COL1A2 Mutations in Osteogenesis Imperfecta.** *J Mol Diagn* 2011, **13:**648-656.
5. Tappino B, Chuzhanova NA, Regis S, Dardis A, Corsolini F, Stroppiano M, Tonoli E, Beccari T, Rosano C, Mucha J, Blanco M, Szlago M, Di Rocco M, Cooper DN, Filocamo M: Molecular **characterization of 22 novel UDP-N-acetylglucosamine-1-phosphate transferase alpha- and beta-subunit (GNPTAB) gene mutations causing mucolipidosis types II alpha/beta and III alpha/beta in 46 patients.** *Hum Mutat* 2009, **30:**E956-E973.
6. Porto C, Cardone M, Fontana F, Rossi B, Tuzzi MR, Tarallo A, Barone MV, Andria G, Parenti G: **The pharmacological chaperone N-butyldeoxynojirimycin enhances enzyme replacement therapy in Pompe disease fibroblasts.** *Mol Ther* 2009, **17:**964-971.
7. Santamaria R, Michelakakis H, Moraitou M, Dimitriou E, Dominissini S, Grossi S, Sánchez-Ollé G, Chabás A, Pittis MG, Filocamo M, Vilageliu L, Grinberg D: **Haplotype analysis suggests a single Balkan origin for the Gaucher disease [D409H;H255Q] double mutant allele.** *Hum Mutat* 2008, **29:**E58-67.
8. Caiazzo M, Dell'anno MT, Dvoretskova E, Lazarevic D, Taverna S, Leo D, Sotnikova TD, Menegon A, Roncaglia P, Colciago G, Russo G, Carninci P, Pezzoli G, Gainetdinov RR, Gustincich S, Dityatev A, Broccoli V: **Direct generation of functional dopaminergic neurons from mouse and human fibroblasts.** *Nature* 2011, **476:**224-227.
9. Nikkel SM, Dauber A, de Munnik S, Connolly M, Hood RL, Caluseriu O, Hurst J, Kini U, Nowaczyk MJ, Afenjar A, Albrecht B, Allanson JE, Balestri P, Ben-Omran T, Brancati F, Cordeiro I, da Cunha BS, Delaney LA, Destrée A, Fitzpatrick D, Forzano F, Ghali N, Gillies G, Harwood K, Hendriks YM, Héron D, Hoischen A, Honey EM, Hoefsloot LH, Ibrahim J, et al.: **The phenotype of floating-harbor syndrome: clinical characterization of 52 individuals with mutations in exon 34 of SRCAP.** *Orphanet J Rare Dis* 2013, **8:**63.
10. De Vita S, Canzonetta C, Mulligan C, Delom F, Groet J, Baldo C, Vanes L, Dagna-Bricarelli F, Hoischen A, Veltman J, Fisher EM, Tybulewicz VL, Nizetic D: **Trisomic dose of several chromosome 21 genes perturbs haematopoietic stem and progenitor cell differentiation in Down's syndrome.** *Oncogene* 2010, **29:**6102-6114.
11. Sessarego N, Parodi A, Podestà M, Benvenuto F, Mogni M, Raviolo V, Lituania M, Kunkl A, Ferlazzo G, Bricarelli FD, Uccelli A, Frassoni F: **Multipotent mesenchymal stromal cells from amniotic fluid: solid perspectives for clinical application.** *Haematologica* 2008, **93:**339-346.
12. Colombo EA, Bazan JF, Negri G, Gervasini C, Elcioglu NH, Yucelten D, Altunay I, Cetincelik U, Teti A, Del Fattore A, Luciani M, Sullivan SK, Yan AC, Volpi L, Larizza L: **Novel C16ORF57 mutations in patients with Poikiloderma with neutropenia: bioinformatic analysis of the protein and predicted effects of all reported mutations.** *Orphanet J Rare Dis* 2012, **7**:7.
13. Passerini L, Di Nunzio S, Gregori S, Gambineri E, Cecconi M, Seidel MG, Cazzola G, Perroni L, Tommasini A, Vignola S, Guidi L, Roncarolo MG, Bacchetta R: **Functional type 1 regulatory t cells develop regardless of FOXP3 mutations in patients with Ipex syndrome.** *Eur J Immunol* 2011, **41:**1120-1131.
14. Madia F, Striano P, Di Bonavebntura C, De falc A, De falco F, Manfredi M, Striano S, Minetti M, Zara F. **Bening adult familial myoclonic epilepsy (BAFME): evidence of an extended founder haplotype on chromosome 2p11.1-q12.2 in five italian families.** *Neurogenetics* 2008, **9:**139-142.
15. Teodelinda M, Michele C, Sebastiano C, Ranieri C, Chiara G: **Amniotic liquid derived stem cells as reservoir of secreted angiogenic factors capable of stimulating neo-arteriogenesis in an ischemic model.** *Biomaterials* 2011, **32:**3689-99.
16. Cilia R, Kraff J, Canesi M, Pezzoli G, Goldwurm S, Amiri K, Tang HT, Pan R, Hagerman PJ, Tassone F: **Screening for the presence of FMR1 Premutation Alleles in Females with Parkinsonism.** *Arch Neurol* 2009, **66:**244-249.
17. Lautier C, Goldwurm S, Dürr A, Giovannone B, Tsiaras WG, Pezzoli G, Brice A, Smith RJ: **Mutations in the GIGYF2 Gene at the PARK11 Locus in Familial Parkinson's Disease.** *Am J Hum Gen* 2008, **82:**822-833.
18. Cartelli D, Goldwurm S, Casagrande F, Pezzoli G, Cappelletti G**: Microtubule destabilization is shared by genetic and idiopathic Parkinson's disease patient fibroblasts.** *PLoS One* 2012, **7:**e37467. Erratum in: *PLoS One* 2012, **7.**
19. Healy DG, Falchi M, O'Sullivan SS, Bonifati V, Durr A, Bressman S, Brice A, Aasly J, Zabetian CP, Goldwurm S, Ferreira JJ, Tolosa E, Kay DM, Klein C, Williams DR, Marras C, Lang AE, Wszolek ZK, Berciano J, Schapira AH, Lynch T, Bhatia KP, Gasser T, Lees AJ, Wood NW; on behalf of the International LRRK2 Consortium: **Phenotype, genotype, and worldwide genetic penetrance of LRRK2-associated Parkinson's disease: a case-control study.** *Lancet Neurol* 2008, **7:**583-590.
20. Höglinger GU, Melhem NM, Dickson DW, Sleiman PM, Wang LS, Klei L, Rademakers R, de Silva R, Litvan I, Riley DE, van Swieten JC, Heutink P, Wszolek ZK, Uitti RJ, Vandrovcova J, Hurtig HI, Gross RG, Maetzler W, Goldwurm S, Tolosa E, Borroni B, Pastor P; PSP Genetics Study Group, Cantwell LB, Han MR, Dillman A, van der Brug MP, Gibbs JR, Cookson MR, Hernandez DG, et al.: **Identification of common variants influencing risk of the tauopathy progressive supranuclear palsy.** *Nat Genet* 2011, **43:**699-705.
21. Liu GH, Qu J, Suzuki K, Nivet E, Li M, Montserrat N, Yi F, Xu X, Ruiz S, Zhang W, Wagner U, Kim A, Ren B, LiY, Goebl A, Kim J, Soligalla RD, Dubova I, Thompson J, Yates J 3rd, Esteban CR, Sancho-Martinez I, Izpisua Belmonte JC: **Progressive degeneration of human neural stem cells caused by pathogenic LRRK2.** *Nature* 2012, **491:**603-607.
22. Ariani F, Mari F, Amitrano S, Di Marco C, Artuso R, Scala E, Meloni I, Della Volpe R, Rossi A, van Bokhoven H, Renieri A: **Exome sequencing overrides formal genetics: ASPM mutations in a case study of apparent X-linked microcephalic intellectual deficit.** *Clin Genet* 2013, **83:**288-290.
23. Ariani F, Hayek G, Rondinella D, Artuso R, Mencarelli MA, Spanhol-Rosseto A, Pollazzon M, Buoni S, Spiga O, Ricciardi S, Meloni I, Longo I, Mari F, Broccoli V, Zappella M, Renieri A: **FOXG1 is responsible for the congenital variant of Rett syndrome.** *Am J Hum Genet* 2008, **83:**89-93.
24. Makrythanasis P, Kapranov P, Bartoloni L, Reymond A, Deutsch S, Guigó R, Denoeud F, Drenkow J, Rossier C, Ariani F, Capra V, Excoffier L, Renieri A, Gingeras TR, Antonarakis SE: **Variation in novel exons (RACEfrags) of the MECP2 gene in Rett syndrome patients and controls.** *Hum Mutat* 2009, **30:**E866-E879.
25. Mencarelli MA, Tassini M, Pollazzon M, Vivi A, Calderisi M, Falco M, Fichera M, Monti L, Buoni S, Mari F, Engelke U, Wevers RA, Hayek J and Renieri A: **Creatine Transporter Defect Diagnosed by ProtonNMR Spectroscopy in Males With Intellectual Disability.** *Am J Med Genet A* 2011, **155A:**2446-2452.
26. De Filippis R, Pancrazi L, Bjørgo K, Rosseto A, Kleefstra T, Grillo E, Panighini A, Cardarelli F, Meloni I, Ariani F, Mencarelli MA, Hayek J, Renieri A, Costa M, Mari F: **Expanding the phenotype associated with FOXG1 mutations and in vivo FoxG1 chromatin-binding dynamics.** *Clin Genet* 2012, **82:**395-403.
27. Bijlsma EK, Collins A, Papa FT, Tejada MI, Wheeler P, Peeters EA, Gijsbers AC, van de Kamp JM, Kriek M, Losekoot M, Broekma AJ, Crolla JA, Pollazzon M, Mucciolo M, Katzaki E, Disciglio V, Ferreri MI, Marozza A, Mencarelli MA, Castagnini C, Dosa L, Ariani F, Mari F, Canitano R, Hayek G, Botella MP, Gener B, Mínguez M, Renieri A, Ruivenkamp CA: **Xq28 duplications including MECP2 in five females: Expanding the phenotype to severe mental retardation.** *Eur J Med Genet* 2012, **55:**404-413.
28. Amenduni M, De Filippis R, Cheung AY, Disciglio V, Epistolato MC, Ariani F, Mari F, Mencarelli MA, Hayek Y, Renieri A, Ellis J, Meloni I: **iPS cells to model CDKL5-related disorders.** *Eur J Hum Genet* 2011, **19):**1246-1255.
29. Fanin M, Nascimbeni AC, Aurino S, Tasca E, Pegoraro E, Nigro V, Angelini C: **Frequency of LGMD gene mutations in Italian patients with distinct clinical phenotypes.** *Neurology* 2009, **72:**1432-1435.
30. Torella A, Fanin M, Mutarelli M, Peterle E, Del Vecchio Blanco F, Rispoli R, Savarese M, Garofalo A, Piluso G, Morandi L, Ricci G, Siciliano G, Angelini C, Nigro V: **Next-Generation Sequencing Identifies Transportin 3 as the Causative Gene for LGMD1F.** *PLoS One* 2013, **8:**e63536.
31. Nascimbeni AC, Fanin M, Masiero E, Angelini C, Sandri M: **The role of autophagy in the pathogenesis of glycogen storage disease type II (GSDII).** *Cell Death Differ* 2012, **19:**1698-1708.
32. Spinazzi M, Casarin A, Pertegato V, Ermani M, Salviati L, Angelini C: **Optimization of respiratory chain enzymatic assays in muscle for the diagnosis of mitochondrial disorders.** *Mitochondrion* 2011, **11:**893-904
33. Nascimbeni AC, Fanin M, Tasca E, Angelini C: **Transcriptional and translational effects of intronic CAPN3 gene mutations.** *Hum Mutat* 2010, **31:**E1658-1669.
34. Angelini C, Semplicini C, Ravaglia S, Moggio M, Comi GP, Musumeci O, Pegoraro E, Tonin P, Filosto M, Servidei S, Morandi L, Crescimanno G, Marrosu G, Siciliano G, Mongini T, Toscano A, Italian Group on GSDII: **New motor outcome function measures in evaluation of late-onset Pompe disease before and after enzyme replacement therapy.** *Muscle Nerve* 2012, **45:**831-834.
35. Fanin M, Benedicenti F, Fritegotto C, Nascimbeni A, Peterle E, Stanzial F, Cristofoletti A, Castellan C, Angelini C: **An intronic mutation causes severe LGMD2A in a large inbred family belonging to a genetic isolate in the Alps.** *Clin Genet* 2012, **82:**601-602.
36. Mancuso M, Angelini C, Bertini E, Carelli V, Comi GP, Minetti C, Moggio M, Mongini T, Servidei S, Tonin P, Toscano A, Uziel G, Zeviani M, Siciliano G; Nation-wide Italian Collaborative Network of Mitochondrial Diseases: **Fatigue and exercise intolerance in mitochondrial diseases. Literature revision and experience of the Italian Network of mitochondrial diseases.** *Neuromuscul Disord* 2012, **22(**Suppl 3):S226-S229.
37. Kornblum C, Nicholls TJ, Haack TB, Schöler S, Peeva V, Danhauser K, Hallmann K, Zsurka G, Rorbach J, Iuso A, Wieland T, Sciacco M, Ronchi D, Comi GP, Moggio M, Quinzii CM, DiMauro S, Calvo SE, Mootha VK, Klopstock T, Strom TM, Meitinger T, Minczuk M, Kunz WS, Prokisch H: **Loss-of-function mutations in MGME1 impair mtDNA replication and cause multisystemic mitochondrial disease.** *Nat Genet* 2013, **45:**214-219.
38. Ronchi D, Di Fonzo A, Lin W, Bordoni A, Liu C, Fassone E, Pagliarani S, Rizzuti M, Zheng L, Filosto M, Ferrò MT, Ranieri M, Magri F, Peverelli L, Li H, Yuan YC, Corti S, Sciacco M, Moggio M, Bresolin N, Shen B, Comi GP: **Mutations in DNA2 link progressive myopathy to mitochondrial DNA instability.** *Am J Hum Genet* 2013, **92:**293-300.
39. Ronchi D, Garone C, Bordoni A, Gutierrez Rios P, Calvo SE, Ripolone M, Ranieri M, Rizzuti M, Villa L, Magri F, Corti S, Bresolin N, Mootha VK, Moggio M, Dimauro S, Comi GP, Sciacco M: **Next-generation sequencing reveals DGUOK mutations in adult patients with mitochondrial DNA multiple deletions.** *Brain* 2012, **135:**3404-3415.
40. Magri F, Govoni A, D'Angelo MG, Del Bo R, Ghezzi S, Sandra G, Turconi AC, Sciacco M, Ciscato P, Bordoni A, Tedeschi S, Fortunato F, Lucchini V, Bonato S, Lamperti C, Coviello D, Torrente Y, Corti S, Moggio M, Bresolin N, Comi GP: **Genotype and phenotype characterization in a large dystrophinopathic cohort with extended follow-up.** *J Neurol* 2011, **258:**1610-1623.
41. De Palma C, Morisi F, Cheli S, Pambianco S, Cappello V, Vezzoli M, Rovere-Querini P, Moggio M, Ripolone M, Francolini M, Sandri M, Clementi E: **Autophagy as a new therapeutic target in Duchenne muscular dystrophy.** *Cell Death Dis* 2012, **3:**e418.
42. Mercuri E, Messina S, Bruno C, Mora M, Pegoraro E, Comi GP, D'Amico A, Aiello C, Biancheri R, Berardinelli A, Boffi P, Cassandrini D, Laverda A, Moggio M, Morandi L, Moroni I, Pane M, Pezzani R, Pichiecchio A, Pini A, Minetti C, Mongini T, Mottarelli E, Ricci E, Ruggieri A, Saredi S, Scuderi C, Tessa A, Toscano A, Tortorella G, et al.: **Congenital muscular dystrophies with defective glycosylation of dystroglycan: a population study.** *Neurology* 2009, **72:**1802-1809.
43. Saredi S, Ardissone A, Ruggieri A, Mottarelli E, Farina L, Rinaldi R, Silvestri E, Gandioli C, D’Arrigo S, Salerno F, Morandi L, Grammatico P, Pantaleoni C, Moroni I, Mora M: **Novel POMGnT1 point mutations and intragenic rearrangements associated with muscle-eye-brain disease.** *J Neurol Sci* 2012, **318:**45-50.
44. Manzini MC, Tambunan DE, Hill RS, Yu TW, Maynard TM, Heinzen EL, Shianna KV, Stevens CR, Partlow JN, Barry BJ, Rodriguez J, Gupta VA, Al-Qudah AK, Eyaid WM, Friedman JM, Salih MA, Clark R, Moroni I, Mora M, Beggs AH, Gabriel SB, Walsh CA: **Exome sequencing and functional validation in zebrafish identify GTDC2 mutations as a cause of Walker-Warburg syndrome.** *Am J Hum Genet* 2012, **91:**541-547.
45. Cesana M, Cacchiarelli D, Legnini I, Santini T, Sthandier O, Chinappi M, Tramontano A, Bozzoni I: **A long noncoding RNA controls muscle differentiation by functioning as a competing endogenous RNA.** *Cell* 2011, **147:**358-369. Erratum in: *Cell* 2011, **147:**947.
46. Zanotti S, Gibertini S, Savadori P, Mantegazza R, Mora M: **Duchenne muscular dystrophy fibroblast nodules: a cell-based assay for screening anti-fibrotic agents.** *Cell Tissue Res* 2013, **352:**659-670.
47. Mercuri E, Messina S, Bruno C, Mora M, Pegoraro E, Comi GP, D'Amico A, Aiello C, Biancheri R, Berardinelli A, Boffi P, Cassandrini D, Laverda A, Moggio M, Morandi L, Moroni I, Pane M, Pezzani R, Pichiecchio A, Pini A, Minetti C, Mongini T, Mottarelli E, Ricci E, Ruggieri A, Saredi S, Scuderi C, Tessa A, Toscano A, Tortorella G, et al.: **Congenital muscular dystrophies with defective glycosylation of dystroglycan: a population study.** *Neurology* 2009, **72:**1798-1799.
48. Incitti T, De Angelis FG, Cazzella V, Sthandier O, Pinnarò C, Legnini I, Bozzoni I: **Exon skipping and duchenne muscular dystrophy therapy: selection of the most active U1 snRNA antisense able to induce dystrophin exon 51 skipping.** *Mol Ther* 2010, **18:**1675-1682.
49. Guglieri M, Magri F, D’Angelo MG, Prelle A, Morandi L, Rodolico C, Cagliani R, Mora M, Fortunato F, Bordoni A, Del Bo R, Ghezzi S, Pagliarani S, Lucchiari S, Salani S, Zecca C, Lamperti C, Ronchi D, Aguennouz M, Ciscato P, Di Blasi C, Ruggieri A, Moroni I, Turconi A, Moggio M, Toscano A, Bresolin N, P, Comi GP: **Clinical, molecular, and protein correlations in a large sample of genetically diagnosed Italian limb girdle muscular dystrophy patients.** *Hum Mutat* 2008, **29:**258-266.
50. Tedesco FS, Gerli MF, Perani L, Benedetti S, Ungaro F, Cassano M, Antonini S, Tagliafico E, Artusi V, Longa E, Tonlorenzi R, Ragazzi M, Calderazzi G, Hoshiya H, Cappellari O, Mora M, Schoser B, Schneiderat P, Oshimura M, Bottinelli R, Sampaolesi M, Torrente Y, Broccoli V, Cossu G: **Genetic Correction and Transplantation of Human iPSC-Derived Progenitors for Treating Limb-Girdle Muscular Dystrophy.** *Sci Transl Med* 2012, **4:**140ra89.
51. Micale L, Augello B, Fusco C, Selicorni A, Loviglio MN, Silengo MC, Reymond A, Gumiero B, Zucchetti F, D'Addetta EV, Belligni E, Calcagnì A, Digilio MC, Dallapiccola B, Faravelli F, Forzano F, Accadia M, Bonfante A, Clementi M, Daolio C, Douzgou S, Ferrari P, Fischetto R, Garavelli L, Lapi E, Mattina T, Melis D, Patricelli MG, Priolo M, Prontera P, et al.: **Mutation spectrum of MLL2 in a cohort of Kabuki syndrome patients.** *Orphanet J Rare Dis* 2011, **6:**38.
52. Ejarque I, Uliana V, Forzano F, Marciano C, Merla G, Zelante L, Di Maria E, Faravelli F: **Is Hardikar syndrome distinct from Kabuki (Niikawa-Kuroki) sindrome?** *Clinical Genetics* 2011, **80:**493-496.
53. Cacciottolo M, Numitone G, Aurino S, Caserta IR, Fanin M, Politano L, Minetti C, Ricci E, Piluso G, Angelini C, Nigro V: **Muscular dystrophy with marked Dysferlin deficiency is consistently caused by primary dysferlin gene mutations.** *Eur J Hum Genet* 2011, **19:**974-980.
54. Viggiano E, Picillo E, Cirillo A, Politano L: **Comparison of X-chromosome inactivation in Duchenne muscle/myocardium-manifesting carriers, non-manifesting carriers and related daughters.** *Clin Genet* 2012, Oct 30. [Epub ahead of print]
55. Bello L, Piva L, Barp A, Taglia A, Picillo E, Vasco G, Pane M, Previtali SC, Torrente Y, Gazzerro E, Motta MC, Grieco GS, Napolitano S, Magri F, D'Amico A, Astrea G, Messina S, Sframeli M, Vita GL, Boffi P, Mongini T, Ferlini A, Gualandi F, Soraru' G, Ermani M, Vita G, Battini R, Bertini E, Comi GP, Berardinelli A, et al.: **Importance of SPP1 genotype as a covariate in clinical trials in Duchenne muscular dystrophy.** *Neurology* 2012, **79:**159-62.
56. Panteghini C, Zorzi G, Venco P, Dusi S, Reale C, Brunetti D, Chiapparini L, Zibordi F, Siegel B, Garavaglia B, Simonati A, Bertini E, Nardocci N, Tiranti V: **C19orf12 and FA2H Mutations Are Rare in Italian Patients With Neurodegeneration With Brain Iron Accumulation.** *Semin Pediatr Neurol* 2012, **19:**75-81.
57. Haack TB, Hogarth P, Kruer MC, Gregory A, Wieland T, Schwarzmayr T, Graf E, Sanford L, Meyer E, Kara E, Cuno SM, Harik SI, Dandu VH, Nardocci N, Zorzi G, Dunaway T, Tarnopolsky M, Skinner S, Frucht S, Hanspal E, Schrander-Stumpel C, Héron D, Mignot C, Garavaglia B, Bhatia K, Hardy J, Strom TM, Boddaert N, Houlden HH, Kurian MA, et al.: **Exome sequencing reveals de novo WDR45 mutations causing a phenotypically distinct, X-linked dominant form of NBIA.** *Am J Hum Genet* 2012, **91:**1144-1149.
58. Campanella A, Privitera D, Guaraldo M, Rovelli E, Barzaghi C, Garavaglia B, Santambrogio P, Cozzi A, Levi S: **Skin fibroblasts from pantothenate kinase-associated neurodegeneration patients show altered cellular oxidative status and have defective iron-handling properties.** *Hum Mol Genet* 2012, **21:**4049-4059.
59. Leoni V, Strittmatter L, Zorzi G, Zibordi F, Dusi S, Garavaglia B, Venco P, Caccia C, Souza AL, Deik A, Clish CB, Rimoldi M, Ciusani E, Bertini E, Nardocci N, Mootha VK, Tiranti V: **Metabolic consequences of mitochondrial coenzyme A deficiency in patients with PANK2 mutations.** *Mol Genet Metab* 2012, **105:**463-471
60. Chiapparini L, Savoiardo M, D'Arrigo S, Reale C, Zorzi G, Zibordi F, Cordelli DM, Franzoni E, Garavaglia B, Nardocci N: **The "eye-of-the-tiger" sign may be absent in the early stages of classic pantothenate kinase associated neurodegeneration.** *Neuropediatrics* 2011, **42:**159-162.
61. Santorelli FM, Garavaglia B, Cardona F, Nardocci N, Bernardina BD, Sartori S, Suppiej A, Bertini E, Claps D, Battini R, Biancheri R, Filocamo M, Pezzini F, Simonati A: **Molecular epidemiology of childhood neuronal ceroid-lipofuscinosis in Italy.** *Orphanet J Rare Dis* 2013, **8:**19.
